# Identification of an extracellular matrix signature for predicting prognosis and sensitivity to therapy of patients with gastric cancer

**DOI:** 10.1038/s41598-025-88376-8

**Published:** 2025-03-03

**Authors:** Nan Xu, Taojing Zhang, Weiwei Sun, Chenxiao Ye, Huamiao Zhou

**Affiliations:** 1 School of Life Sciences, Zhejiang Chinese Medical University, Hangzhou, 310053 China; 2 The First Clinical Medical College, Zhejiang Chinese Medical University, Hangzhou, 310053 China; 3 Department of Medical Oncology, The First Affiliated Hospital of Zhejiang Chinese Medical University, Hangzhou, 310006 China

**Keywords:** Gastric cancer, Extracellular matrix, Weighted gene co-expression network analysis, Prognosis, Immunotherapy, Cancer, Computational biology and bioinformatics, Immunology

## Abstract

Extracellular matrix (ECM) is a vital component of the tumor microenvironment and plays a crucial role in the development and progression of gastric cancer (GC). Co-expression networks were established by means of the “WGCNA” package, the optimal model for extracellular matrix scores (ECMs) was developed and validated, with its accuracy in predicting the prognosis and treatment sensitivity of GC patients assessed. We performed univariate cox regression analysis [HR = 6.8 ( 3.3–14 ), *p* < 0.001] which demonstrated that ECMs was an independent risk character and perceptibly superior to other factors with further analysis of multivariate Cox regression [HR = 8.68 ( 4.16–18.08 ), *p* < 0.001]. The nomogram, presenting the clinical prognosis model for GC patients, demonstrated accuracy through KM analysis [HR = 3.97 (2.56–6.16), *p* < 0.001] and ROC curves with AUC values of 0.70, 0.72, and 0.72 at 1, 3, and 5 years, respectively. Using the ECMs model, we stratified GC patients into high- and low-risk groups, enabling precise predictions of prognosis and drug sensitivity. This stratification provides a new strategic direction for the personalized treatment of GC.

**Supplementary Information:**

The online version contains supplementary material available at 10.1038/s41598-025-88376-8.

## Introduction

As the third most common cause of cancer-related death worldwide and the sixth most common disease overall, gastric cancer (GC) continues to pose a serious threat^1^. With a 5-year survival rate of roughly 25%, patients diagnosed with GC usually have a poor prognosis because of metastases, recurrent recurrences^2^. Despite the wide variety of medications available, there are still restrictions on available treatment options due to adverse effects, targetability, and drug resistance. For example, savolitinib is a highly selective small molecule inhibitor which can be used to contrapose advanced GC with mesenchymal epithelial transition factor (MET) abnormalities^3,4^, but it probably leads to acquired resistance in patients with MET-amplified GC for the target mechanisms of resistance MET D1228V/N/H and Y1230C mutations or high copy number MET gene amplifications^5,6^. Triplet chemotherapy with cisplatin, 5-fluorouracil, and docetaxel can improve survival but usually causing serious neutropenia and febrile neutropenia^7^. The cooperation between CCDC8 and JMJD2A, along with mechanisms involving ATP7A and histone H2AX, has been implicated in modulating gastric cancer susceptibility and resistance to oxaliplatin-based therapies, despite its efficacy and associated side effects such as polyneuropathy^8–10^. What’s more, it’s reported that drug resistance is present in 80% of the GC patients, including patients who are administered afatinib or erlotinib, which are important clinical drug^11–13^. Obviously, it’s difficult to achieve ideal effects merely taking conventional treatments. Therefore, identifying new biomarkers is crucial for advancing precision treatment and improving prognostic assessments for GC patients.

The tumor microenvironment (TME), which has long been linked to tumor growth, immune escape, and the efficacy of immunotherapy^14,15^, is made up of cancer cells, stromal cells, blood vessels, nerve fibers, extracellular matrix (ECM), and related acellular components^16^. Numerous research has suggested that expounding the cellular composition stands a chance of providing prognostic information and further improving the applicability of immunotherapy^17,18^. Thus, it’s necessary to conduct a further analysis to complex environment of TME by bioinformatics, which will be conducive to improve the personalized immunotherapy for GC. Therein, ECM, a three-dimensional, non-cellular and highly dynamic structure, is a complex aggregation which mainly consists of collagen, fibrous proteins, proteoglycans, cytokines, and other biomolecule^19,20^. ECM is also a momentous factor dictating tumor destiny^21^. Current evidence has indicated that ECM can lead up various cellular signaling cascades to encourage tumor growth and metastasis by interacting with ambient cells^22^. According to the clinical observation, changes in the composition of the ECM have much to do with invasiveness of tumors and prognosis^23^. Research has shown that epithelial-mesenchymal transition (EMT) is a critical process of tumor metastasis while increased ECM stiffness promotes EMT by activating EPHA2/LYN/TWIST1 signaling cascade in breast cancer^24,25^. In colorectal cancer, it’s confirmed that integrin α2β1 from ECM can boost metastatic capability and stemness of colorectal cancer cells with PI3K/AKT signal activated, besides, as the downstream of PI3K/AKT signaling, snail can further enhance invasion and metastasis of colorectal cancer^26,27^. However, there is a lack of research on evaluating GC with ECM, so it is crucial to investigate the relationship between ECM and GC in order to investigate ECM-based prognostic and efficacy prediction models for GC. These models would be especially beneficial for patient prognostic assessment and the creation of customized treatment strategies.

Thus, we used WGCNA to determine the key modules that are most pertinent to ECM and stromal abundance in GC in light of this creative thinking in this study. Furthermore, we established an ECM signature prognosis model in GC with its activity to assess the prognoses of GC patients precisely and response to immunotherapy evaluated.

## Results

### Identification of ECM-nominated genes

All four cohorts (TCGA-STAD, GSE26942, MSigDB, and GSE184198) were processed to meet the requirements of this study. Missing values and outliers were identified and addressed, while duplicate records were checked and removed. The “ESTIMATE” package was used to evaluate the Stromal Score, Immune Score, and ESTIMATE Score for all specimens in the TCGA-STAD cohort, providing metrics for WGCNA module filtering.

**Fig. 1:**
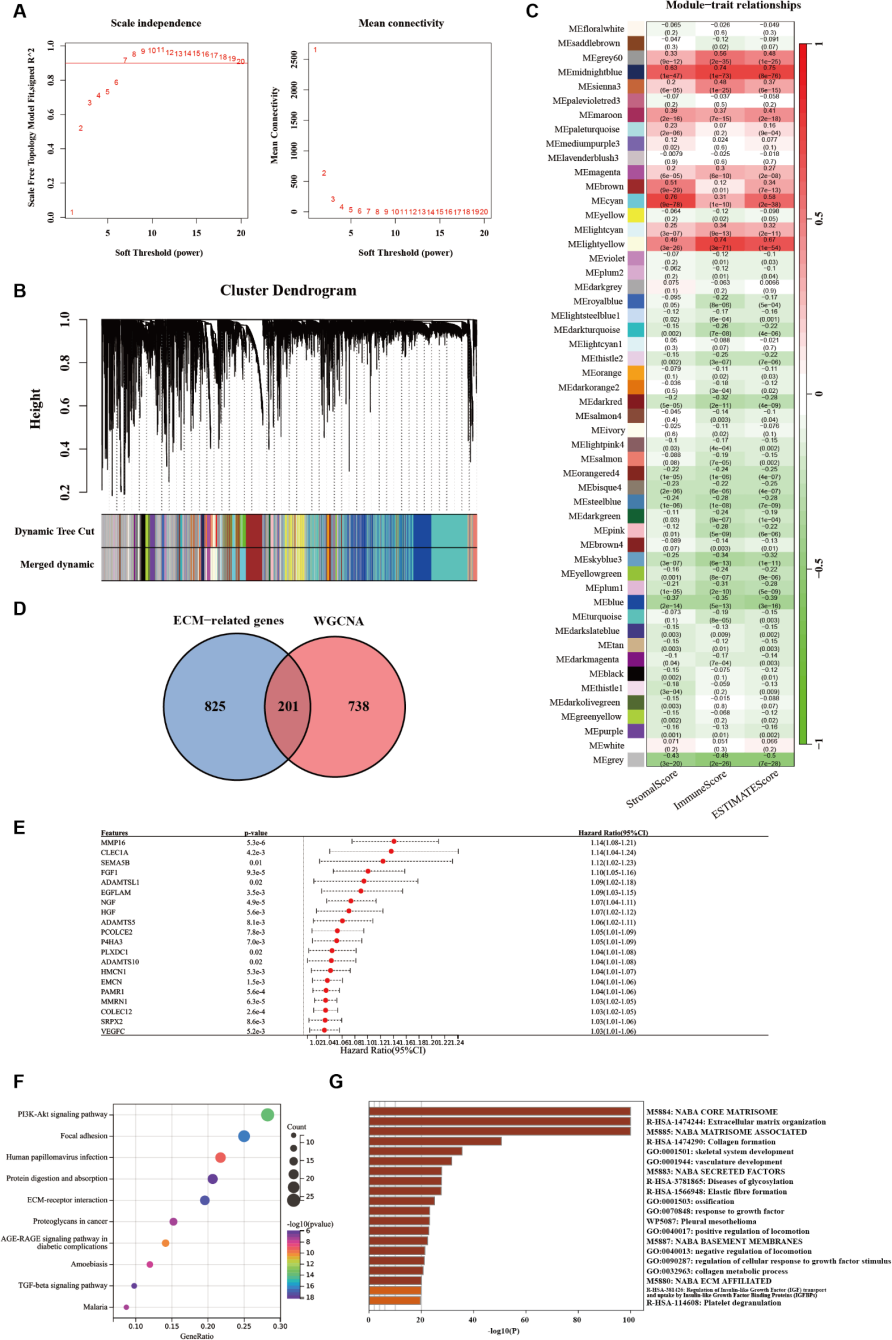
Identification of ECM-nominated Genes. (**A**) The scale-free topology criterion for TCGA-STAD requires a soft threshold power (β) of option 7. (**B**) Gene Co-expression Module Analysis recognized by cluster dendrogram. (**C**) Relevancy analysis of modules with StromalScore, ImmuneScore and ESTIMATEScore. (**D**) Venn diagram of the 1,026 ECM genes and 937 ECM-associated genes from WGCNA. (**E**) Forest plots presenting univariate Cox regression analyses of candidate ECM genes as independent prognostic factors. (**F**, **G**) Enrichment analysis of the candidate ECM genes by virtue of KEGG Enrichment Analysis (**F**) and Metascape database (**G**), respectively.

Based on the clinical data, the optimal Soft Threshold value was determined to be 7 (Fig. 1A), resulting in the generation of a clustering dendrogram that identified 55 modules (Fig. 1B). Among these, a specific and significant module, designated as cyan, was identified with high correlations to the Stromal Score (0.76), Immune Score (0.31), and ESTIMATE Score (0.58), all with p-values < 0.0001 (Fig. 1C). These findings indicate that this module is highly associated with the extracellular matrix (ECM) and immune infiltration. Further analysis using the MSigDB cohort yielded 201 genes from this module (Fig. 1D), which were subsequently categorized as ECM-related genes.

To refine these candidate genes, univariate Cox regression analysis was conducted to assess both the Hazard Ratio (HR) and p-values, identifying 83 significant genes, with the top 20 listed (Fig. 1E). Further exploration of these genes revealed that ECM-related genes were primarily enriched in the PI3K-Akt signaling pathway, as indicated by KEGG^28–30^ enrichment analysis (Fig. 1F), and in the NABA CORE MATRISOME category, based on the Metascape database (Fig. 1G).

### The establishment of ECMs-score scoring model and prognostic analysis

**Fig. 2:**
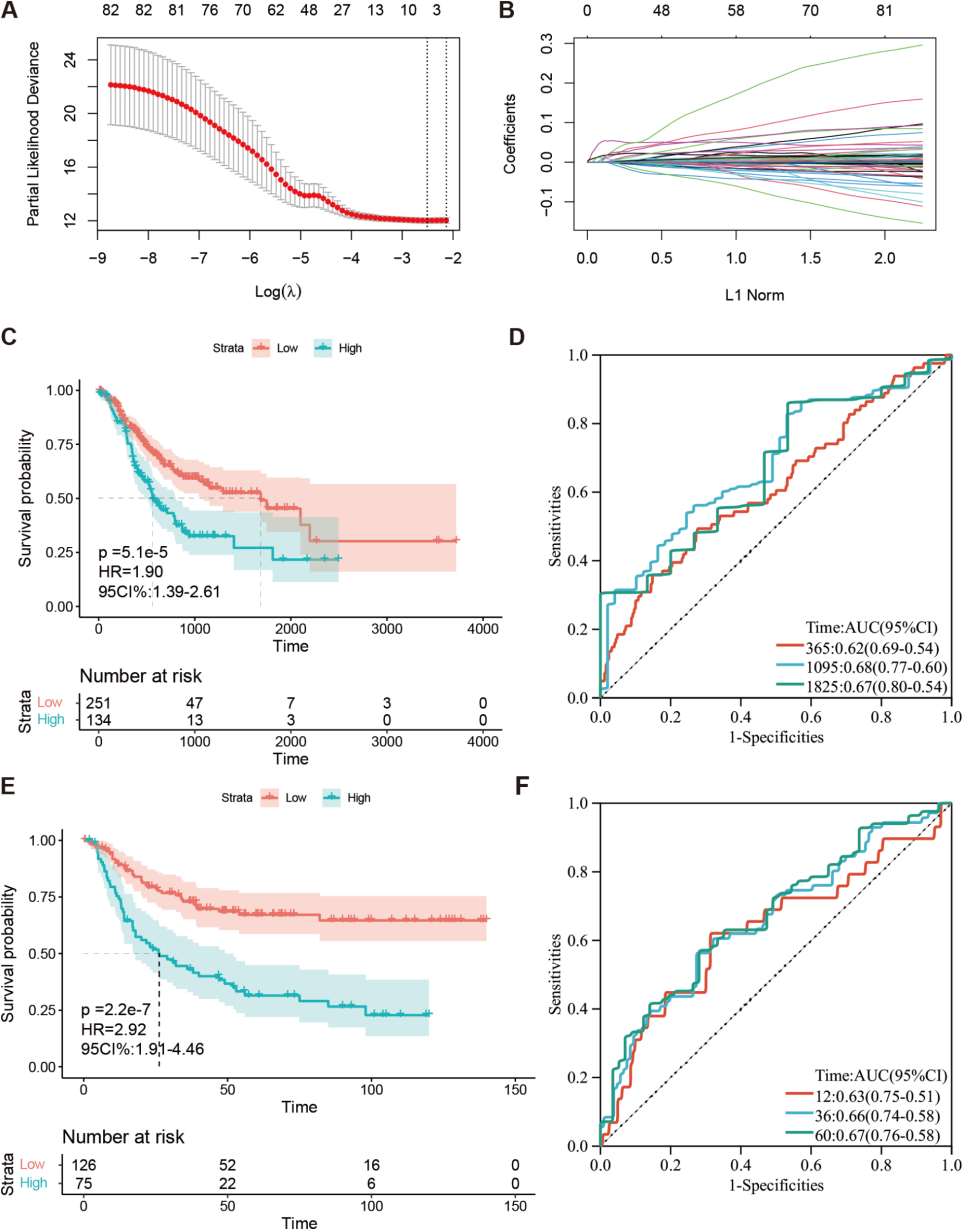
Establishment of ECMs-Score Scoring Model. (**A**, **B**) Coefficient profiles (**B**) of LASSO-Cox regression analysis, and the adjustment parameter (lambda) was calculated based on the partial likelihood deviance with ten-fold cross validation (**A**). (**C**, **E**) Kaplan-Meier analyses determined GC patients in the high-ECMs-risk group exhibiting worse overall survival both in TCGA-STAD (**C**) and GSE26942 (**E**) cohort. (**D**, **F**) ROC curves of ECMS in TCGA-STAD (**D**) and GSE26942 (**F**) cohort.

The candidate genes filtered through univariate analysis underwent further testing using LASSO-Cox regression analysis (Fig. 2A and B), resulting in the identification of six key genes (Table S1) for the construction of the ECMs risk score model, with a λ value of 0.068. The final ECMs risk score was calculated as follows: ECMs risk score = expression of MMP16 * 0.04 + expression of NGF * 0.01 + expression of MMRN1 * 0.012 + expression of ANXA5 * 0.0006 + expression of IGFBP7 * 8.5e-06 + expression of PLOD2 * 0.00013. With that, it was eventually demonstrated that the model made sense in virtue of Kaplan-Meier (KM) curves [HR = 1.90 ( 1.39–2.61 ), *p* < 0.001 in the training cohort (Fig. 2C), HR = 2.92 ( 1.91–4.46 ), *p* < 0.001 in the validation (Fig. 2E) ]. Further confirmation was provided by Receiver Operating Characteristic (ROC) curve analysis, which yielded AUC values of 0.62, 0.68, and 0.67 at 1, 3, and 5 years, respectively, for the training cohort (Fig. 2D) and AUC values of 0.63, 0.66, and 0.67 at the same time points for the validation cohort (Fig. 2F), underscoring the model’s accuracy.

**Fig. 3:**
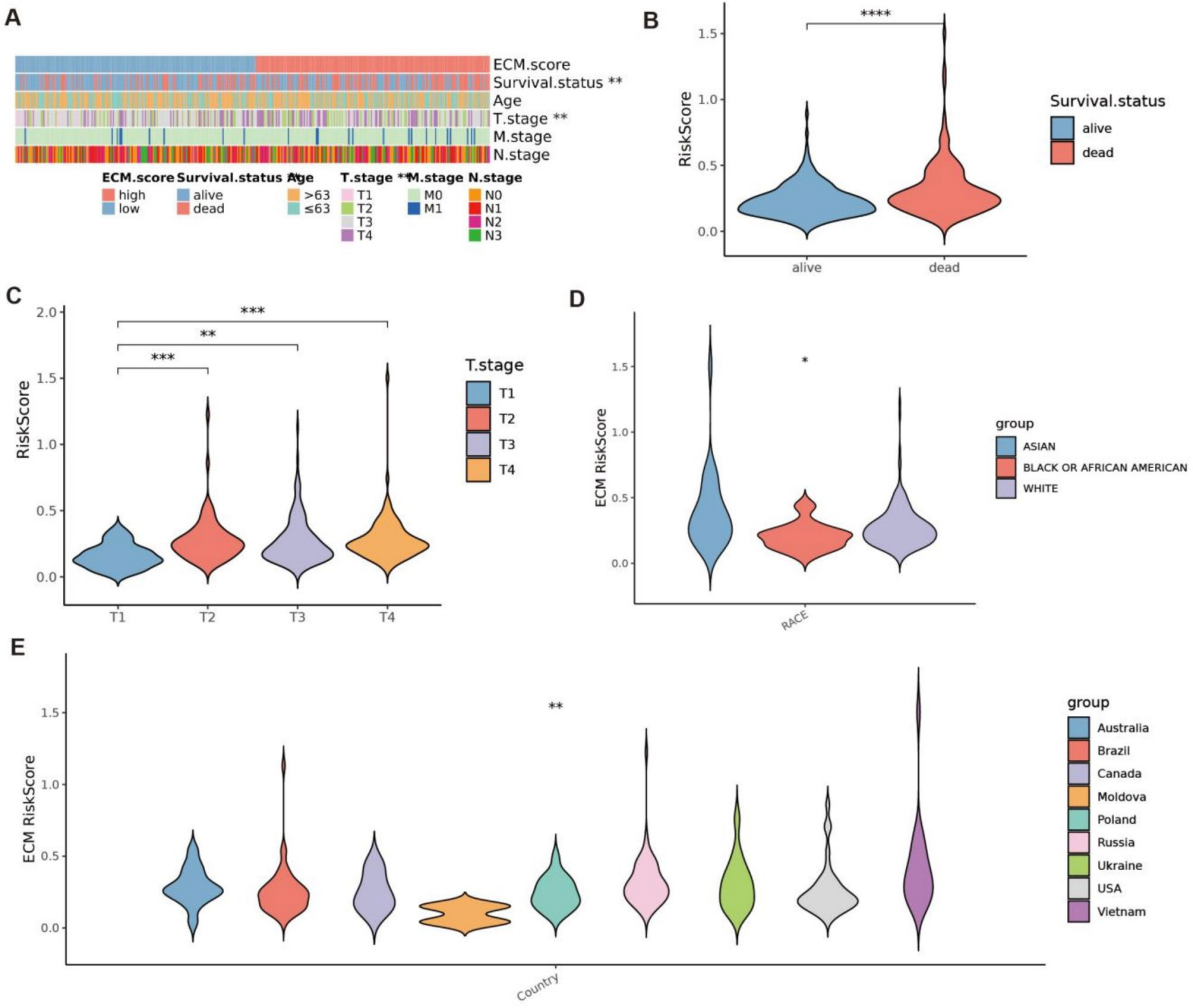
Correlations between ECMs and clinical characteristics in GC. (**A**) Heatmap of the clinicopathological characteristics and ECMs. (**B**, **C**) The ECMs in different groups classified based on clinical characteristics from TCGA-STAD. (**D**, **E**) Violin diagrams of geographically and ethnically diverse cohorts and ECMs from TCGA-STAD. *p < 0.05, **p < 0.01, ***p < 0.001, ****p < 0.0001.

Next, we analyzed the clinical characteristics of patients in the low- and high-ECM risk score groups, including 5-year survival rates, age, T stage, N stage, and M stage (Fig. 3A). Significant differences were observed in the distributions of these clinical characteristics between the two groups. Consistent with our previous findings, the high-ECM risk score group exhibited a significantly lower 5-year survival rate (Fig. 3B). Additionally, a higher ECM risk score was strongly associated with a more advanced T stage (Fig. 3C). These findings suggest that the ECM risk score is closely linked to a higher risk of poor clinical outcomes.

To further evaluate the robustness and generalizability of our model, we conducted additional analyses using geographically and ethnically diverse cohorts from TCGA-STAD (Fig. 3D-E). These analyses revealed significant differences in ECM-related characteristics among GC patients from different ethnicities and countries, underscoring the broader applicability of our findings.

**Fig. 4:**
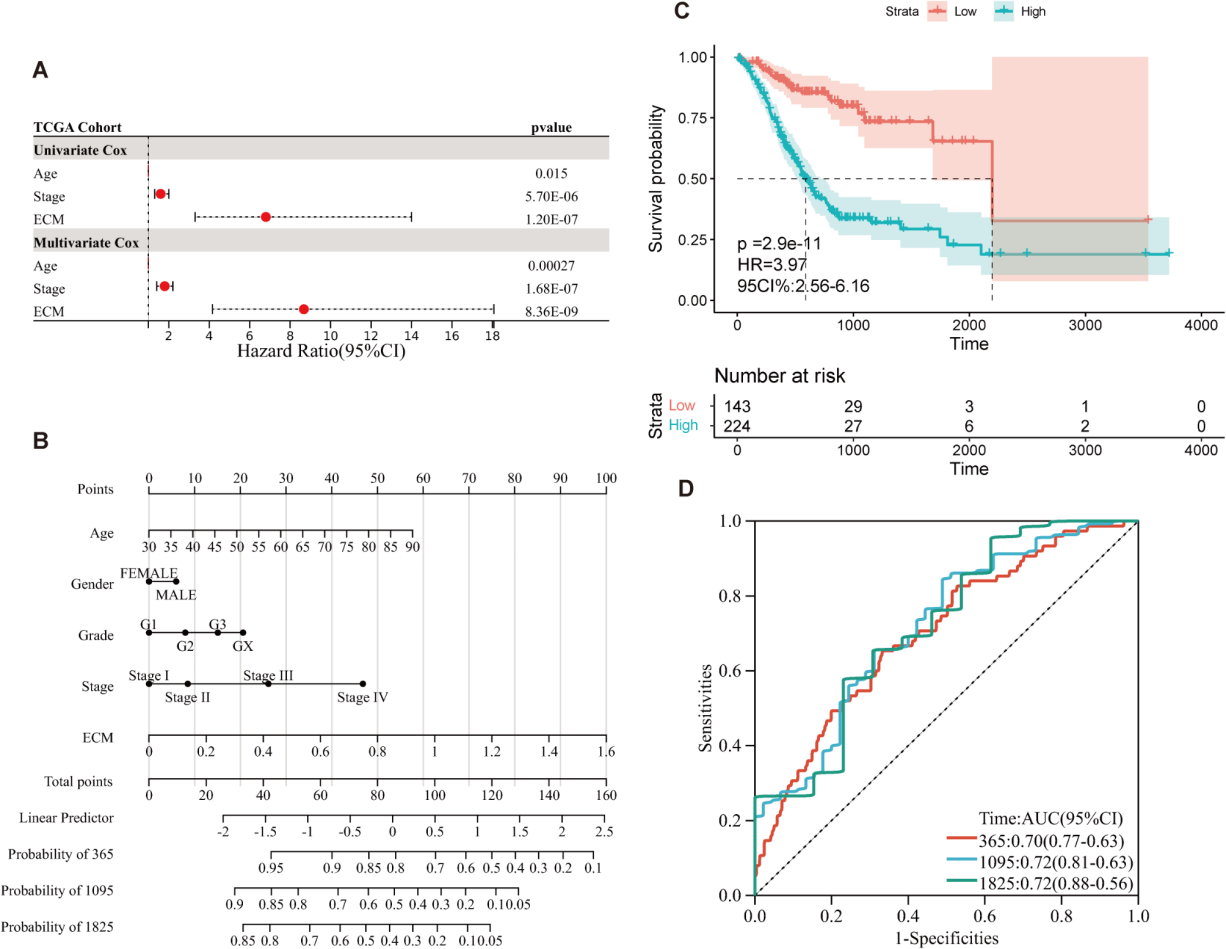
Prognostic Analysis of ECMs. (**A**) Forest plots of univariate regression analyses and multivariable regression analyses, the ECM, Age, and Stage contained, indicating that ECM is an independent prognostic predictor and superior to other features. (**B**) Nomogram showing the risk assessment of patients. (**C**, **D**) the KM curves (**C**) and ROC curves (**D**) of Nomogram calibration predicting the OS at 1, 3, and 5 years.

We further explored the relationship between age, clinical stage, ECMs, and prognosis through univariate Cox regression analysis, which indicated that ECMs was an independent risk factor for prognosis [HR = 6.8 (3.3–14), *p* < 0.001; Fig. 4A]. This result demonstrated that ECMs was a stronger predictor of survival compared to other clinical factors. Multivariate Cox regression analysis reinforced these findings, indicating that ECMs maintained its prognostic significance [HR = 8.68 (4.16–18.08), *p* < 0.001; Fig. 4A].

To improve the clinical application of the ECMs model, we developed a nomogram that combines clinical and ECMs risk score data to predict the survival probability of GC patients (Fig. 4B). KM analysis confirmed the prognostic value of the nomogram, with HR = 3.97 (2.56–6.16), *p* < 0.001 (Fig. 4C), and ROC curve analysis yielded AUC values of 0.70, 0.72, and 0.72 at 1, 3, and 5 years, respectively (Fig. 4D), further validating the accuracy of the nomogram in predicting patient outcomes.

### Functional enrichment analysis

**Fig. 5:**
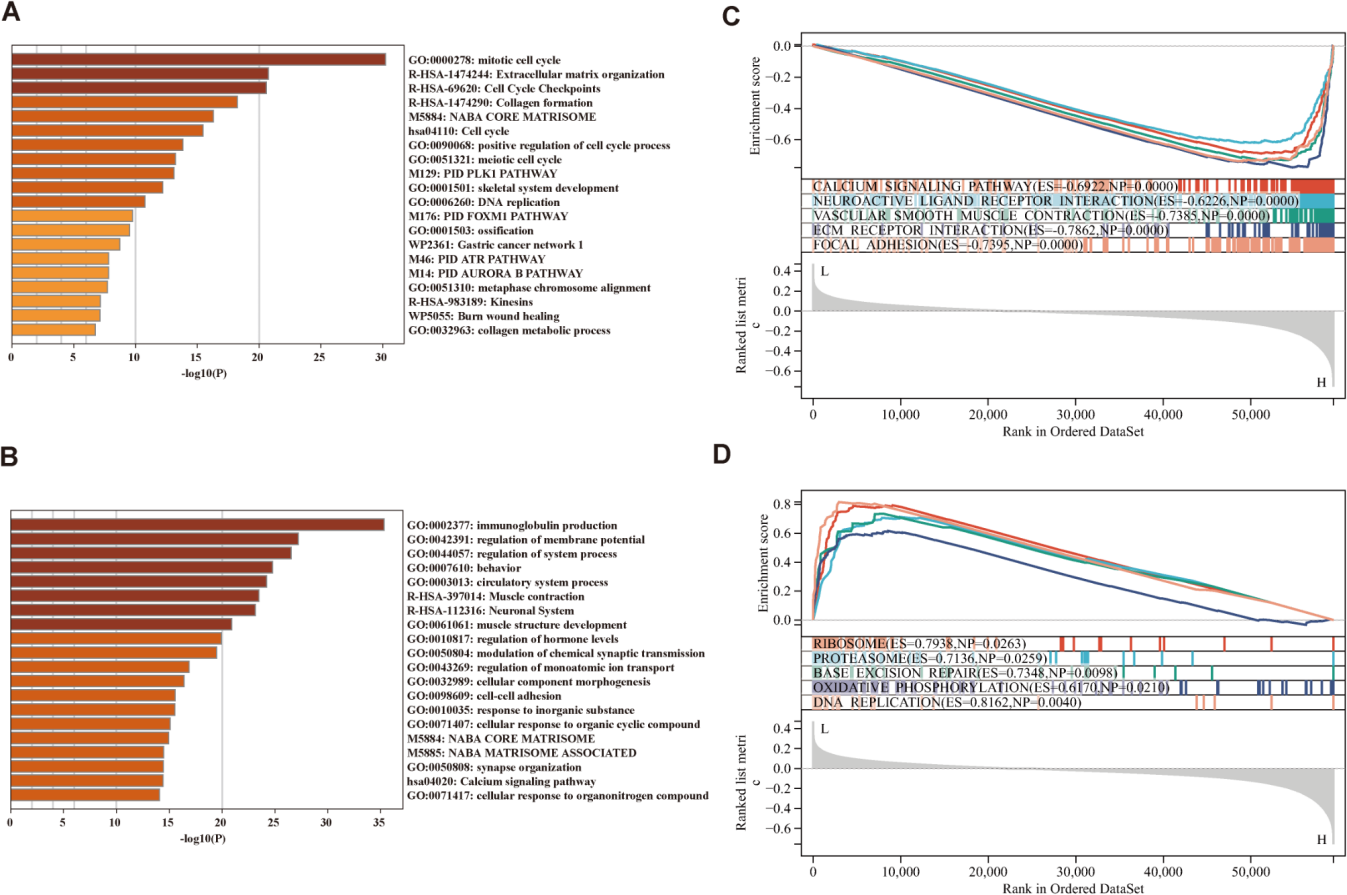
Functional enrichment analysis. (**A**, **B**) Enrichment analysis of the DEGs in the High (**A**) and the Low (**B**) groups through Metascape database; (**C**, **D**) GSEA of KEGG gene sets between the High (**C**) and the Low (**D**) groups.

To explore the underlying mechanisms by which ECMs characteristics influence the prognosis of GC, we performed a functional enrichment analysis using ssGSEA to analyze the GO and KEGG gene sets between the high- and low-ECM tisk groups, according to the optimum truncation. Differentially expressed genes (DEGs) were identified using the “limma” package, and the results were submitted to Metascape for further analysis. The results revealed that genes upregulated in the high ECM group were significantly associated with processes such as the mitotic cell cycle, ECM organization, and cell cycle checkpoints (Fig. 5A). In contrast, genes downregulated in the low ECM group were closely related to immunoglobulin production, regulation of membrane potential, and system process regulation (Fig. 5B).

Further analysis using Gene Set Enrichment Analysis (GSEA) with gene expression data from the high and low ECM groups confirmed these findings. The DEGs were sorted by fold change to illustrate the trends of gene expression differences between the two groups. Functional enrichment analysis of the high ECM group showed that DEGs were predominantly involved in pathways such as Calcium Signaling, Neuroactive Ligand Receptor Interaction, Vascular Smooth Muscle Contraction, ECM Receptor Interaction, and Focal Adhesion (Fig. 5C). In contrast, the DEGs in the low ECM group were mainly enriched in pathways related to Ribosome, Proteasome, Base Excision Repair, Oxidative Phosphorylation, and DNA Replication (Fig. 5D).

Additional validation of these findings revealed consistent enrichment patterns. In the high ECM group, the DEGs were primarily associated with pathways such as Glycosphingolipid Biosynthesis Ganglio Series, Glycosaminoglycan Biosynthesis Chondroitin Sulfate, ECM Receptor Interaction, Primary Bile Acid Biosynthesis, and Vascular Smooth Muscle Contraction (Figure S1A). For the low ECM group, the dominant pathways included Steroid Biosynthesis, DNA Replication, Homologous Recombination, Proteasome, and Base Excision Repair (Figure S1B).

### Immunoinfiltration analysis and prediction of drug susceptibility response

**Fig. 6:**
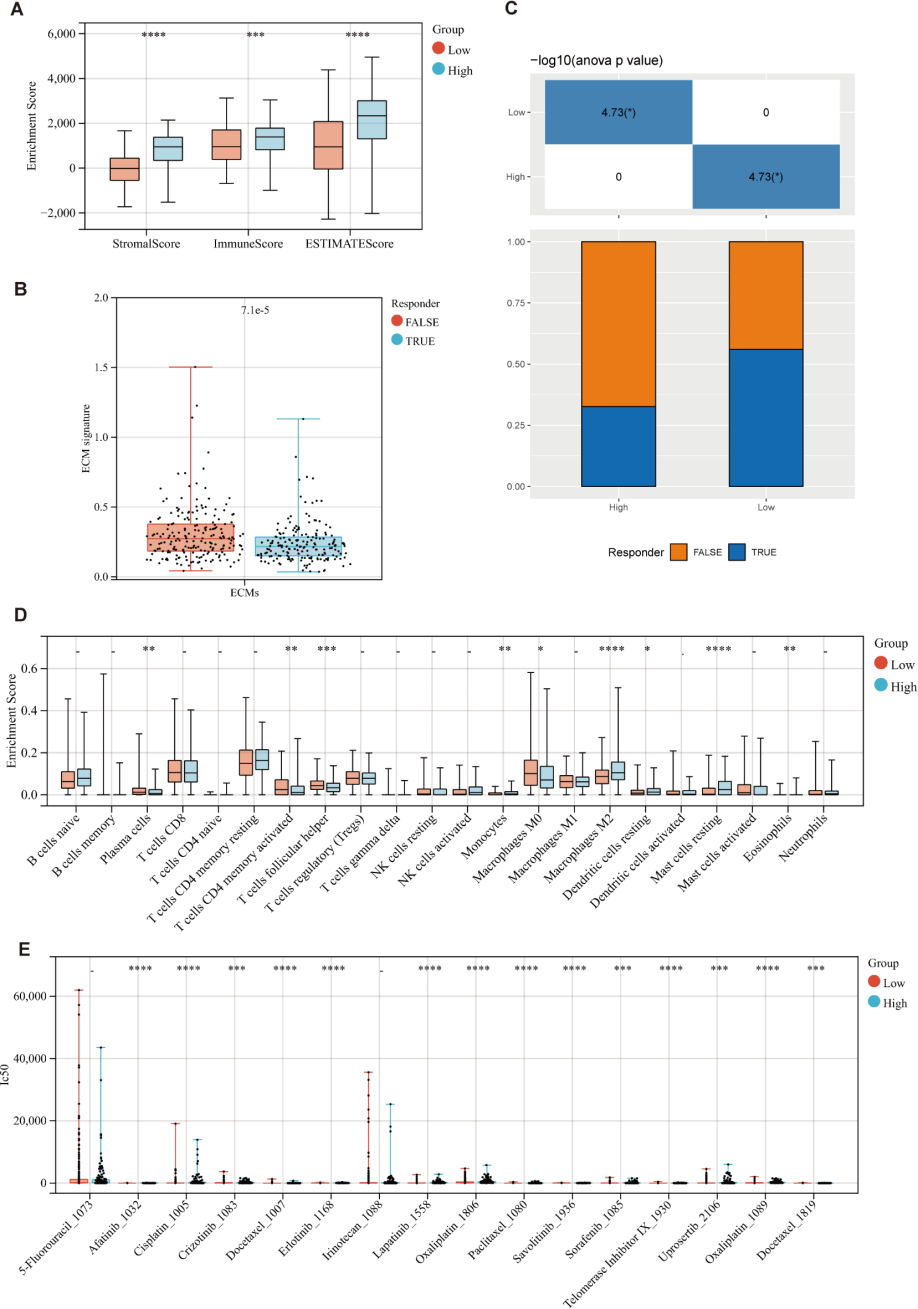
Immune landscape and Prediction of drug susceptibility response. (**A**) Box diagram of the “ESTIMATE” arithmetic score. (**B**, **C**) TIDE score predicts response to immunotherapy. The ECM signature discrepancies between immunotherapy-responders and non-responders groups. (**B**); Distributions of responders and non-responders in high- and low- ECMs-risk groups (**C**). (**D**)) Box diagram of the proportion of immune cell infiltration. (**E**) Box diagram comparing IC50 values of several chemotherapy drugs between high- and low- ECMs-risk groups. *p < 0.05; **p < 0.01; ***p < 0.001; ****p < 0.0001; ns, no significance.

The Estimate score, based on the ssGSEA algorithm, showed significant differences in StromalScore, ImmuneScore, and ESTIMATEScore between the high and low ECM risk groups (*p* < 0.001). The relative abundance of mesenchymal and immune cells in the high ECM risk group was notably higher than in the low ECM risk group, with significant differences observed in immune cell infiltration and immune response levels (Fig. 6A). This highlights the strong correlation of our ECM model with the relative abundance of immune cells in tumor samples.

At the same time, we applied the TIDE method to assess whether the ECM risk score could serve as a predictor for immunotherapy response in GC patients. For the TCGA-STAD cohort, the ECM risk score in the non-response subgroup was significantly higher than in the response group (*p* < 0.001; Fig. 6B). GC patients in the low ECM risk group were more sensitive to immunotherapy (140/251) compared to those in the high ECM risk group (44/134) (*p* < 0.05; Fig. 6C), suggesting that our ECM model is effective in predicting immunotherapy response.

Furthermore, CIBERSORTx was used to analyze the fraction of 22 immune-infiltrating cell types in patient samples. Significant differences in the distribution of 22 infiltrating immune cells between the high and low ECM risk groups were observed (Fig. 6D), with notable differences in nine subtypes of immune cells (*p* < 0.05). Four subtypes were downregulated in the high ECM risk group, including plasma cells (*p* < 0.01), activated memory CD4 + T cells (*p* < 0.01), follicular helper T cells (*p* < 0.001), and M0 macrophages (*p* < 0.05). Conversely, five cell types were upregulated in the high ECM risk group, including monocytes (*p* < 0.01), M2 macrophages (*p* < 0.0001), resting dendritic cells (*p* < 0.05), resting mast cells (*p* < 0.05), and eosinophils (*p* < 0.01).

Based on the GDSC database, Wilcoxon analysis revealed significant differences in the IC50 values of commonly used GC drugs between patients with high and low ECM risk. Patients in the low ECM risk group were more sensitive to afatinib, cisplatin, crizotinib, docetaxel, erlotinib, lapatinib, oxaliplatin, savolitinib, sorafenib, telomerase inhibitor IX, uprosertib, and docetaxel (*p* < 0.001). On the other hand, the high ECM risk group showed greater sensitivity to paclitaxel (*p* < 0.001). The evaluation of 5-Fluorouracil and Irinotecan showed no significant differences due to outliers (Fig. 6E). These findings underscore the ECM model’s potential in drug susceptibility assessment, offering a new dimension for personalized treatment strategies.

### Substantiate the expression of hub genes in GC tissues

Given the findings from CIBERSORT, which indicated a close relationship between ECMs and immune cells, we employed Single-cell Sequencing^31^ to further explore this connection (Figure S2A-D). Interestingly, the six hub genes all exhibited significant expression in tissue stem cells. Specifically, NGF was predominantly expressed in epithelial cells, ANXA5 in monocytes, MMRN1 and IGFBP7 in endothelial cells, and MMP16 in both epithelial and endothelial cells (Figure S2E). These findings suggest that the hub genes play crucial roles in the TME.

**Fig. 7:**
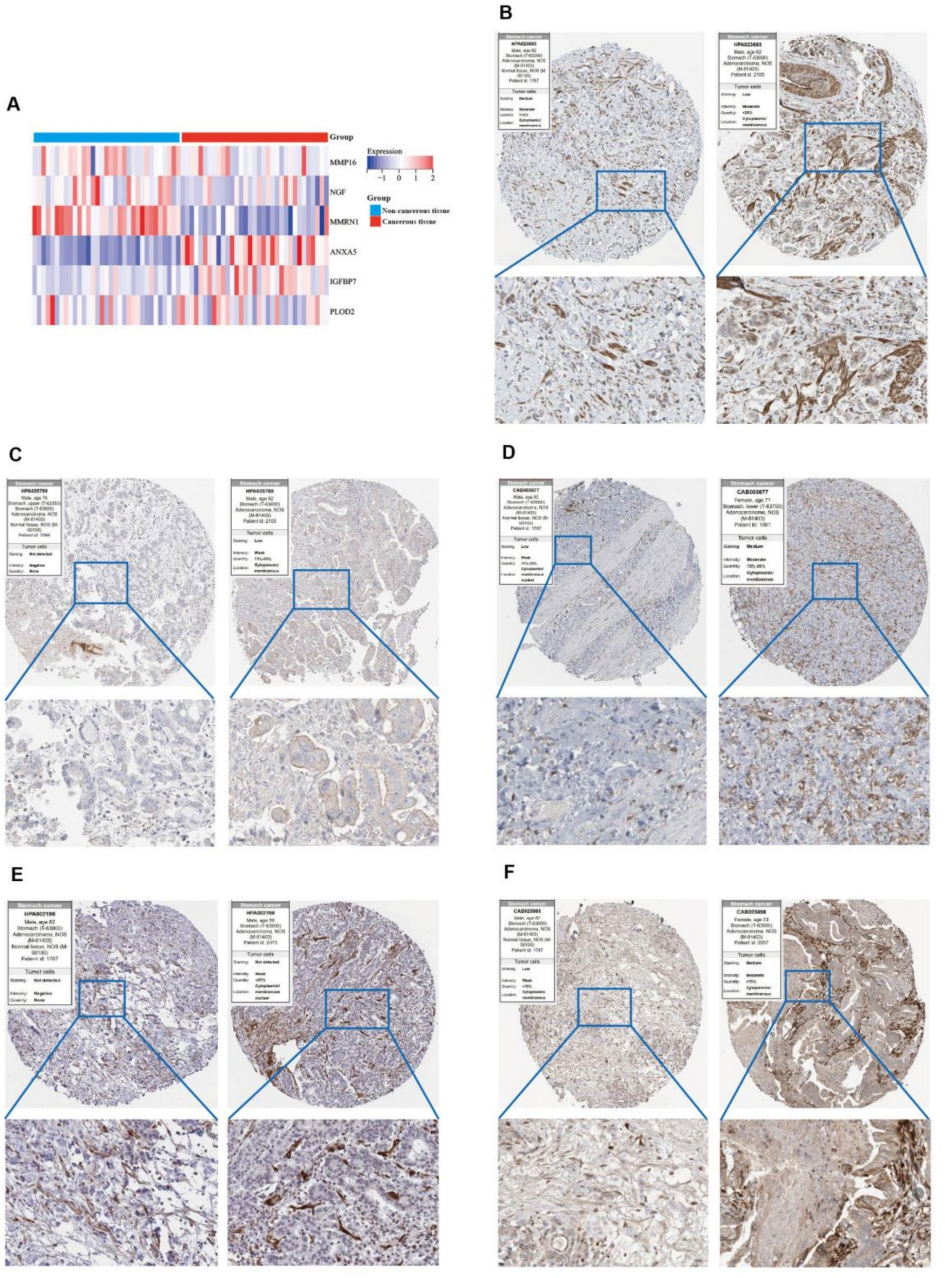
Substantiate the expression of hub genes in GC tissues. (**A**) The heatmap of the hub genes in Non-cancer tissues and Cancerous tissue, respectively. (**B**-**F**) Protein expression levels and expression features of MMP16, MMRN1, ANXA5, IGFBP7 and PLOD2 in normal tissues and gastric cancer specimens from the Human Protein Atlas database.

Based on KM analysis derived from optimal truncation of the hub genes, we observed that patients with high expression of NGF and ANXA5 had poorer outcomes compared to those with low expression of these genes (Figure S3A-F). A heatmap of the six hub genes from TCGA-STAD further illustrates their expression levels in both non-cancerous and cancerous tissues at the mRNA level (Fig. 7A). To determine the protein expression levels of these six ECM-related genes in normal versus tumor tissue, we analyzed IHC images from the HPA database. The results revealed that these proteins were upregulated in tumors and exhibited strong staining in the GC matrix (Fig. 7B-F), though NGF was not included in the database. These findings support the notion that the six hub genes may serve as ECM-specific markers, with NGF requiring further validation in future studies.

## Discussion

In GC, ECM plays a critical role, with its involvement spanning nearly all stages of the disease, from initiation to progression and metastasis^19^. For example, increased expression of tenascin has been observed in precancerous cells^32,33^, while dysregulated collagen expression is evident in more advanced stages of the disease^34^. Consistently, our findings indicate that patients in the high-ECM subgroup exhibit a poorer prognosis, with significant differences in the protein expression levels of ECM-related genes between normal and cancerous gastric tissues. These results suggest that ECM components have substantial clinical potential as prognostic biomarkers and therapeutic targets in GC. This study is the first to employ weighted gene co-expression network analysis (WGCNA) alongside multiple computational algorithms to investigate the ESTIMATE co-expression network of ECM-related genes. By applying univariate Cox regression and the LASSO-Cox regression algorithm, we identified six hub genes that formed a prognostic ECM model. The accuracy of this model was validated using KM analyses and ROC curves. Furthermore, both univariate and multivariate Cox regression analyses, considering age, stage, and ECM scores, confirmed that ECMs serve as independent prognostic factors, outperforming other clinical characteristics. Additionally, we developed a nomogram to assess patient risk, which demonstrated favorable prognostic performance, validated through KM analyses and ROC curves, further supporting its clinical utility.

The TME of GC contains both the ECM and immune cell components such as macrophages, lymphocytes, and neutrophils. These cellular components coexist and interact with each other, and their secreted metabolites and cytokines are as well as significant components of TME. These components of TME in GC play their role in inducing immune tolerance to promote GC progression^14^. Consistently, it is demonstrated synchronously that the ECMs model established is significant for the assessment of immune infiltration, immune checkpoints and immune escape. The immune infiltration analysis reveals a strong correlation between ECMs and various immune cells, especially M2 macrophages, suggesting their interaction with ECM components influences GC progression. Cancer-related metabolic reprogramming, including the acidic TME, plays a pivotal role in malignancy, metastasis, and immune evasion^35^. TME acidosis promotes the polarization of tumor-associated macrophages (TAMs) to the M2 phenotype, enhancing immune suppression^36^. ECMs also serve as reservoirs for TGF-β, which, through mutations in TGF-β signaling molecules, is involved in tumor formation. Tumor-derived TGF-β induces immune suppression by converting T cells to regulatory T cells (Tregs) and polarizing macrophages to the M2 phenotype^37^. TAMs, key players in tumor angiogenesis and lymphangiogenesis, secrete factors like VEGF, bFGF, and MMPs, and also contribute to tumor progression by inhibiting dendritic cell maturation^38,39^. This complex interplay between ECMs, immune cells, and tumor microenvironment significantly influences GC progression. The functional enrichment analysis showed that the candidate ECM genes were mostly enriched in PI3K-Akt signaling pathway which has been implicated in apoptosis, autophagy, and survival in GC^40^. Investigate thoroughly the differential genes in the High and the Low, it presented that the high group of ECM characteristics is strongly interrelated with the cell cycle, meanwhile, the genes down-regulated in low ECM group suggested a considerable association with immunity. The GSEA manifested that the pathways bound with ECM straightway or indirectly affect the occurrence, growth, invasion, and migration of carcinoma. For example, about the Ca subtypes, TRPV2 and TRPV4 are viewed as remarks of prognosis of patients of GC^41^. The ECM receptors exert their function by binding to the ECM ligands, mainly by stimulating integrin-dependent signaling, which promotes invasion and proliferation, creating a favorable microenvironment for metastatic cells, and interfering with communication between tumor cells and immune cells and other pathways^19^. DNA replication is regarded as the important portion of cellular response while uncontrolled DNA replication is one of the typical signs acquired by cancer cells^42,43^. In proteasome, colorectal neoplasia differentially expressed can promote the degradation of serine and arginine-rich splicing factor 6, which plays a role in regulating the sensitivity of GC to drugs^44,45^, and the tripartite motif 69 can inhibit the anoikis resistance and metastasis of GC by mediating the degradation of protein kinase C delta through the ubiquitin-proteasome pathway^46^.

GC treatment typically follows a comprehensive approach, combining radical surgery with adjuvant chemotherapy. The ECM model has been rigorously validated and provides a useful reference for evaluating the drug sensitivity of individual GC patients to commonly used drugs in clinical practice. 5-Fluorouracil (5-FU) is widely used in cancer treatment^47^. However, gastric carcinoma cells have developed resistance to 5-FU due to increasing methylation of genes and proteins, limiting its clinical application^48^. This drug resistance issue is also observed with Cisplatin, Lapatinib, and other chemotherapy agents^49–51^. Patient heterogeneity adds to the complexity of treatment, as individual sensitivities to drugs vary. Our analysis revealed that GC patients with low ECM risk scores exhibited higher sensitivity to treatments with Afatinib, Cisplatin, Crizotinib, Docetaxel, Erlotinib, Lapatinib, Oxaliplatin, Savolitinib, Sorafenib, Telomerase Inhibitor IX, Uprosertib, and Docetaxel. In contrast, subgroups with high ECM scores were more sensitive to Paclitaxel. Evaluation of 5-FU and Irinotecan showed no significant correlation due to outliers, but the reduction of ECM components, such as capillaries and stromal cells, alongside enhanced immune processes, was identified as a key feature of a favorable response to 5-FU, particularly in patients with diffuse GC^52^. Oxaliplatin exposure induces GC cells to secrete ECM, increasing tissue stiffness and promoting mitochondrial transfer via microvesicles from mesenchymal stem cells. These mitochondria fuse with GC cell mitochondria, reducing autophagy and enhancing cell survival. Single-cell sequencing revealed that both GC cells and mesenchymal stem cells actively remodel TME, driving ECM stiffness and drug resistance^53^. Therapeutic strategies targeting ECM remodeling, such as integrin inhibitors or ECM-degrading enzymes, show promise in overcoming these barriers^19^. Furthermore, combining ECM modulation with immune checkpoint inhibitors or anti-angiogenic therapies may enhance treatment efficacy by improving drug penetration and immune cell infiltration in resistant tumors^54^.

Regarding the six discerned genes in the model, ANXA5 can promote GC by affecting ECM-related processes and immune infiltration^55^ with studies suggesting that ANXA5 is not only closely associated with tumorigenesis and progression through PI3K/Akt/NF-κB pathway, but also enhances immunogenicity of tumor cells and the efficacy of related chemotherapy drugs^56–58^. Elevating IGFBP7 level promotes tumor progression by enhancing TAM/M2 macrophage polarization through the FGF2/FGFR1/PI3K/AKT axis in GC^59,60^. PLOD2 encodes a membrane-bound homo-dimeric enzyme helping to form ECM in tumors^61^, which can increase invasiveness and resistance to 5-FU of GC cells^62^. It has been pointed out that NSCLC metastasizes through the EGFR-PI3K/AKT-FOXA1-PLOD2 pathway, providing a basis for PLOD2 as a therapeutic target for NSCLC treatment^63^. It is reported that NGF recruits sensory nerves to the TME through axonal production^64,65^, and promotes the proliferation and growth of tumors using PLC*γ* pathways^66^, possessing the capability to impact ECM by enhancing the proliferation of basal cells^67^. In regard to GC, MMRN1 can interact with platelets to promotes the evasion of circulating cancer cells from natural killer cells, recruit ECM and granulocytes, and release angiogenic growth factors, thereby leading to the occurrence and metastasis of GC^68^. MMP16 is the downstream gene of β-catenin, which is closely associated with Wnt-mediated invasive and metastasis in GC cell^69,70^. It also can activate pro-Matrix Metalloproteinase 2 inducing the denaturation of type IV collagen and partially degrade type I collagen and other ECM proteins in the basement membrane^70^. Simultaneously, the expression of IGFBP7, ANXA5, and PLOD2 was significantly up-regulated in GC, while the expression of MMRN1 genes was prominently down-regulated^71–73^. Both ANXA5 and MMRN1 are considered biomarkers for GC diagnosis and progression^68,73^, while ANXA5 and MMP16 exhibit potential as predictive markers for lymph node and distant organ metastasis^74,75^. However, the exact functions of these ECM markers in GC remain unclear, and further studies are needed to elucidate their roles in proliferation, invasion, and metastasis.

Despite the promising findings, there are still several noteworthy limitations to our study. First, as a retrospective bioinformatics analysis based on three publicly available gene expression datasets, the prognostic accuracy and therapeutic value of the ECMs model require further validation with large sample sizes in real clinical settings. Additionally, the specific biological roles of ECM signature biomarkers in GC need to be confirmed through molecular biology and animal experiments. A key challenge in translating bioinformatics findings into clinical practice lies in patient heterogeneity and dataset biases, which must be addressed in future research. Furthermore, there is a lack of robust experimental methods to study ECM, and developing suitable techniques to explore its role in tumorigenesis and progression is crucial. Despite these limitations, our findings provide a foundational framework for future studies on the role of ECM in GC and its potential as a therapeutic target.

## Conclusion

In summary, we established an ECMs model consisting of six hub genes and classified GC patients into high- and low-ECMs-risk groups to predict the prognosis and drug sensitivity of GC patients, which might provide new insights and options for individualized treatment of GC patients.

## Methods

### Data acquisition and processing

RNA-seq data and clinical information for 448 TCGA stomach adenocarcinoma (TCGA-STAD) cases were obtained from the GDC Data Portal of the Cancer Genome Atlas (TCGA) (https://portal.gdc.cancer.gov/). This dataset includes 412 tumor samples and 36 normal samples. The raw data were normalized to transcripts per million (TPM) and log-transformed. GSE26942^76^, which consists of 202 tumor samples, served as the validation cohort. Additionally, 1026 ECM-related genes were gathered from the Molecular Signatures Database (MSigDB) (https://www.gseamsigdb.org/gsea/msigdb/). These datasets were selected based on the following criteria: (a) primary human gastric cancer samples, (b) intact gene expression matrix, and (c) relevant clinical data including prognosis, stage, gender, and age. Single-cell RNA-seq data containing both GC and normal tissue samples were obtained from GSE184198^77^.

### Weighted gene co-expression network analysis

The “ESTIMATE” package, based on the ssGSEA algorithm, was used to calculate Stromal, Immune, and ESTIMATE Scores for each sample. Co-expression networks were constructed using the “WGCNA” package. We determined the optimal soft threshold for the network by selecting a minimum power that yielded a scale-free topology fit close to 0.9. The co-expression network was then constructed using weighted genes with similar expression profiles, and gene modules were identified. A hierarchical clustering dendrogram was generated after calculating the topological overlap matrix (TOM), with average agglomerative clustering using the median non-weighted pairwise method.

### Prognostic model construction and validation

The intersection of ECM-related genes and those identified through WGCNA was used to create a preliminary set of ECM genes. A prognostic model was constructed using univariate Cox regression analysis, followed by refinement using a false discovery rate (FDR) threshold of < 0.05. The model was further optimized by selecting the λ (lambda.min) that minimized the error during 10-fold cross-validation.

Extracellular matrix scores (ECMs)=

$$\:\sum\:_{k=1}^{n}{\beta\:}_{k}{P}_{k}$$

where k, β_k_, P_k_ refer to the number of dominating kernel genes, the coefficient index, and the gene expression levels, respectively. Patients were then stratified into high- and low-ECM risk groups (henceforth referred to as High and Low, respectively) based on the calculated ECM risk score and the optimal cutoff value. To evaluate the predictive accuracy and robustness of the model, both the training and validation cohorts were analyzed using the “survival” and “survivalROC” R packages. These analyses provided a comprehensive assessment of the model’s prognostic performance and credibility.

### Enrichment analysis and immune landscape

Enrichment Analysis respectively coursed and construed via “clusterProfiler” and “org.Hs.eg.db” packages and Metascape database. As the “limma” package made a thorough inquiry about the differential genes, it was adopted that “clusterProfiler”, “enrichplot” and “patchwork” packages were employed to proceed Gene Set Enrichment Analysis (GSEA) to deliberate the discrepancy genes both in the High and Low groups in which pathway of major enrichment. The immune cell abundance analysis was performed using the “CIBERSORT” package, which deconvoluted the immune cell subtype expression matrix. Tumor immune dysfunction and rejection were evaluated using the TIDE algorithm to assess immune escape potential. The “Predict Response” function was used to evaluate responses to immune checkpoint blockade.

### Drug susceptibility assessment

Drug sensitivity (IC50) was assessed using the “get_oncePredict_res” function from the “oncoPredict” package^77^. Expression data and pType data from the GDSC2 database were obtained from OSFHOME (https://osf.io/), which includes both training sets and corresponding drug response data. The “calcPhenoty” function from the “oncoPredict” package was used to predict the drug activity of each sample, enabling the evaluation of ECM classification effects on clinical drug therapy in GC.

### Human protein atlas (HPA) databases

For protein level analysis, immunohistochemical (IHC) staining images from GC tissues were retrieved from the HPA online database (https://www.proteinatlas.org/)^78^, which yielded the localization and degree of expression of the target proteins.

### Statistical analysis

All statistical analyses and graphing were performed using R software (version 4.3.1). The “WGCNA” package was used to identify co-expressed gene modules, and the “survival,” “survminer,” and “glmnet” packages were employed to construct the prognostic model, perform univariate and multivariate Cox regression analyses, and assess predictive accuracy using the “survivalROC” package. The “rms” package was used to plot the nomogram. Statistical significance was considered at *p* < 0.05 unless otherwise specified.

## Electronic supplementary material

Below is the link to the electronic supplementary material.

Supplementary Material 1

## Data Availability

Publicly available datasets were analyzed in this study. These data can be found here: GDC Data Portal of TCGA (https://portal.gdc.cancer.gov/); GEO datasets (https:// www.ncbi.nlm.nih.gov/geo/): GSE26942 and GSE184198.
