# Supplementary material for: Identification of an extracellular matrix signature for predicting prognosis and sensitivity to therapy of patients with gastric cancer
Source: Sci Rep. 2025 Mar 3;15:7464. doi: 10.1038/s41598-025-88376-8 (PMC11876314; doi:10.1038/s41598-025-88376-8)
Supplement: Supplementary file 1 — Supplementary Material 1 [file 41598_2025_88376_MOESM1_ESM.docx]

**Identification of an Extracellular matrix Signature for Predicting Prognosis and Sensitivity to Therapy of patients with** **gastric cancer**

**Nan Xu ^1^, Taojing Zhang ^1^, Weiwei Sun ^1^, Chenxiao Ye ^2^ and Huamiao Zhou ^3, *^**

1. **Supplementary Figures and Tables**

## **1.1** Supplementary **Tables**

| **Gene** | **Full name** |
| --- | --- |
| MMP16 | Matrix Metalloproteinase 16 |
| NGF | Nerve growth factor |
| MMRN1 | Multimerin-1 |
| ANXA5 | Annexin A5 |
| IGFBP7 | Insulin-like growth factor-binding protein 7 |
| PLOD2 | Procollagen-lysine, 2-oxoglutarate 5-dioxygenase 2 |

**Supplementary Table 1.** Detailed information of the 6 ECMS genes.

## **1.2 Supplementary** Figures

**
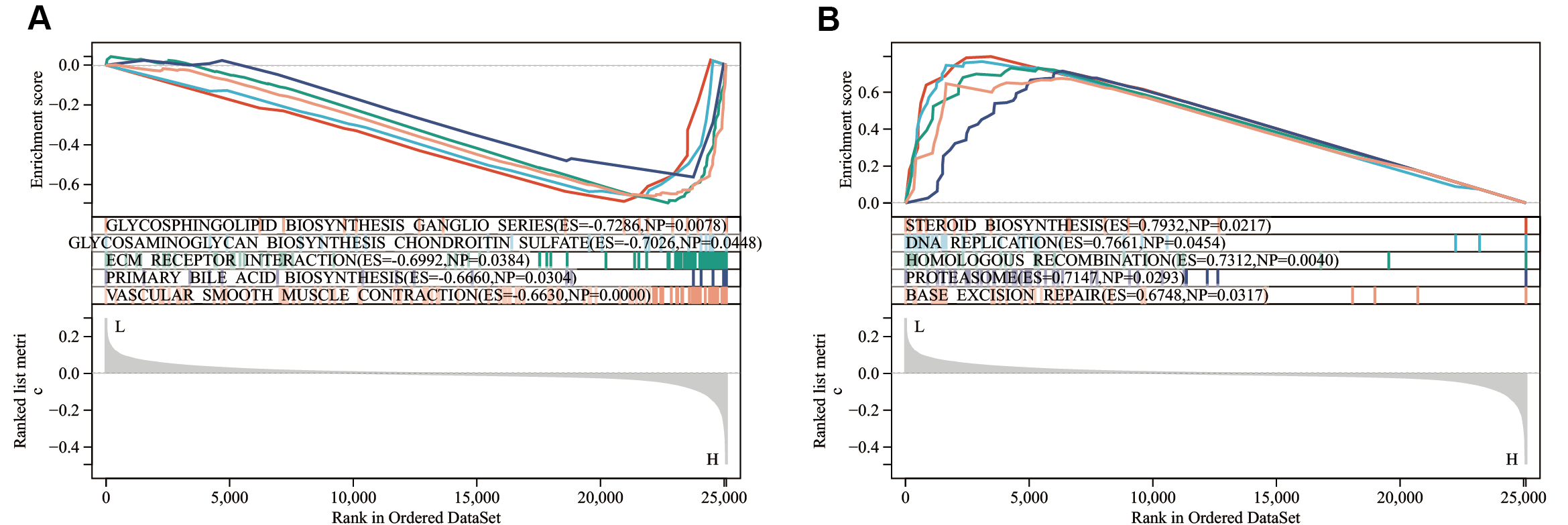
**

**Supplementary Figure 1.** Functional enrichment analysis in GSE26942.


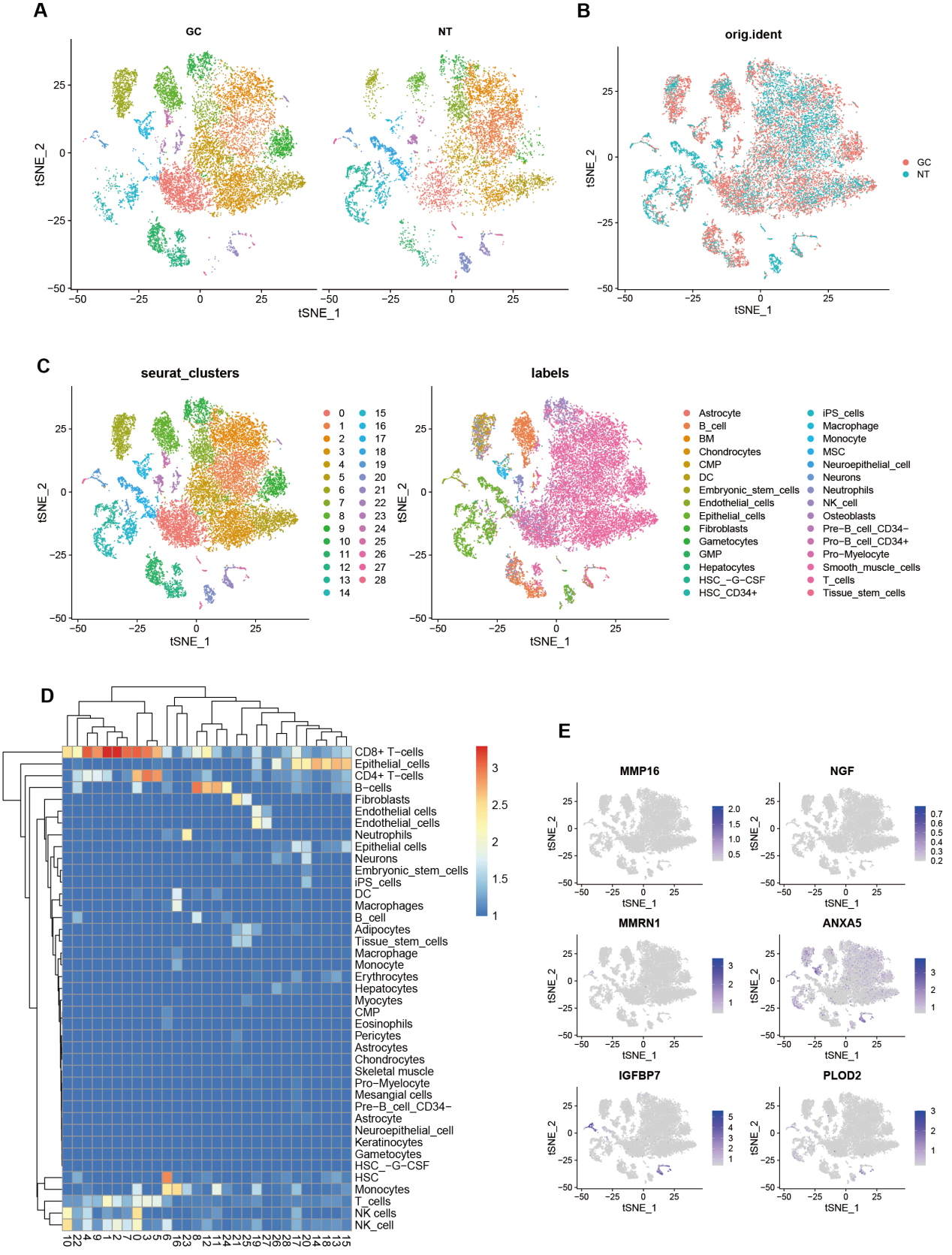


**Supplementary Figure 2.** Comprehensive predictive genes at the single-cell level. (A-B) Integration and differentiation of GC and NT samples. (C-D) Thirty major cell types annotation with automatic-manual inspections: Astrocyte, B cell, BM, Chondrocytes, CMP, DC, Embryonic stem cells, Endothelial cells, Epithelial cells, Fibroblasts, Gametocytes, GMP, Hepatocytes, HSC-G-CSF, HSC CD34^+^, iPS cells, Macrophage, Monocyte, MSC, Neuroepithelial cell, Neurons, Neutrophils, NK cell, Osteoblasts, Pre-B cell CD34^-^, Pro-B cell CD34^+^, Pro-Myelocyte, Smooth muscle cells, T cells, Tissue stem cells. (E) Distribution of 6 hub genes in various cell subset : MMP16, NGF, MMRN1, ANXA5, IGFBP7, PLOD2.

**
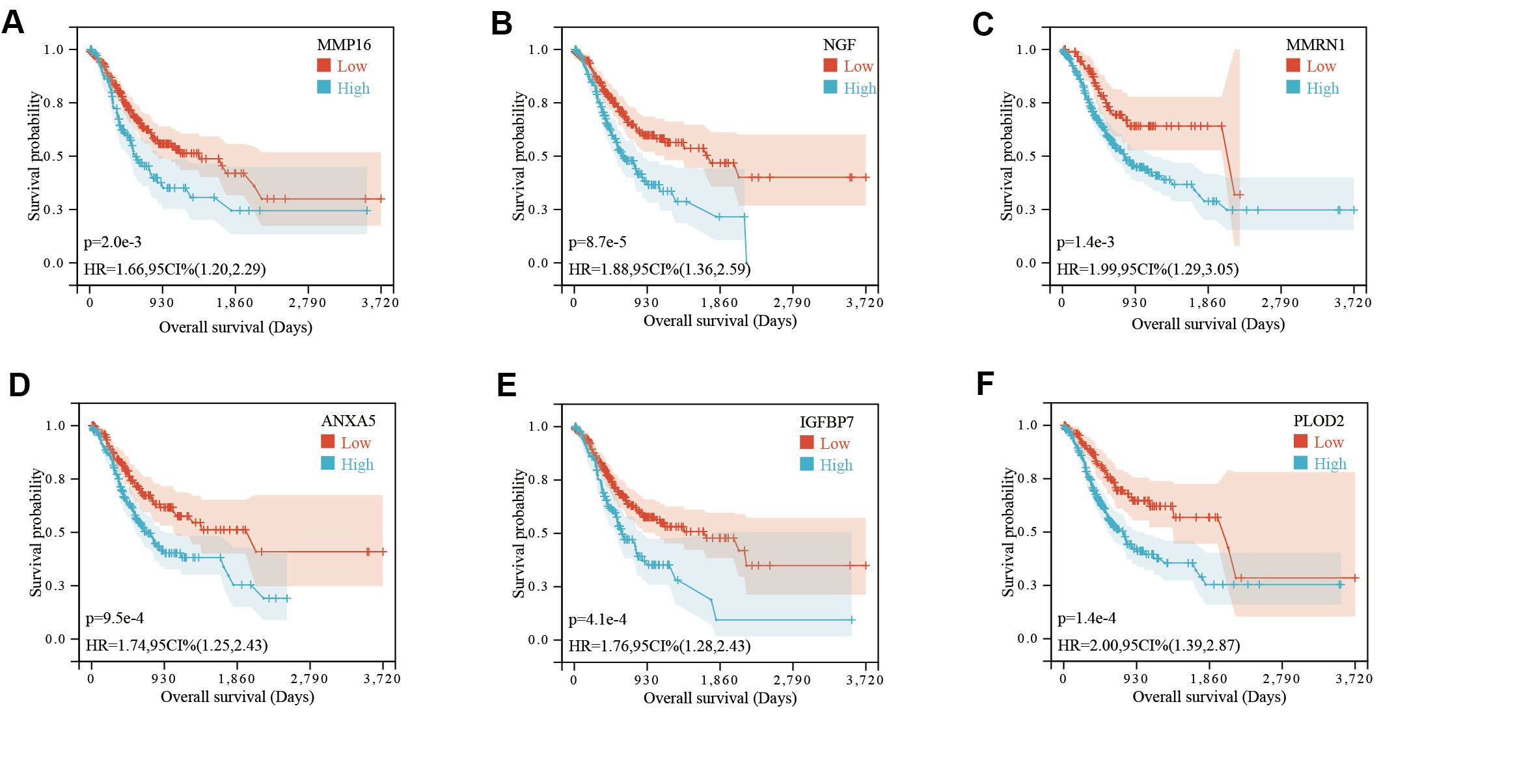
**

**Supplementary Figure 3.** KM analysis of 6 hub genes.
